# Identification and Monitoring of Amomi Fructus and its Adulterants Based on DNA Barcoding Analysis and Designed DNA Markers

**DOI:** 10.3390/molecules24224193

**Published:** 2019-11-19

**Authors:** Eui Jeong Doh, Jung-Hoon Kim, Guemsan Lee

**Affiliations:** 1 Department of Herbology, College of Korean Medicine, Wonkwang University, Iksan 54538, Korea; bluemoonlion@wku.ac.kr; 2 Research Center of Traditional Korean Medicine, Wonkwang University, Iksan 54538, Korea; 3 Division of Pharmacology, School of Korean Medicine, Pusan National University, Yangsan 50612, Korea; kmsct@pusan.ac.kr

**Keywords:** Amomi Fructus, *Amomum villosum*, *Amomum villosum* var. *xanthioide*, *A. longiligulare*, adulterants, DNA barcode, DNA marker, multiplex PCR

## Abstract

Amomi Fructus is one of the traditional medicines derived from the ripe fruits of the Zingiberaceae family of plants, which include *Amomum villosum*, *A. villosum* var. *xanthioides*, and *A. longiligulare*. Owing to their highly similar morphological traits, several kinds of adulterants of Amomi Fructus have been reported. Therefore, accurate and reliable methods of identification are necessary in order to ensure drug safety and quality. We performed DNA barcoding using five regions (ITS, *matK, rbcL, rpoB*, and *trnL-F* intergenic spacer) of 23 Amomi Fructus samples and 22 adulterants. We designed specific DNA markers for Amomi Fructus based on the single nucleotide polymorphisms (SNPs) in the ITS. Amomi Fructus was well separated from the adulterants and was classified with the species of origin based on the detected SNPs from the DNA barcoding results. The AVF1/ISR DNA marker for *A. villosum* produced a 270 bases amplified product, while the ALF1/ISF DNA marker produced a 350 bases product specific for *A. longiligulare*. Using these DNA markers, the monitoring of commercially distributed Amomi Fructus was performed, and the monitoring results were confirmed by ITS analysis. This method identified samples that were from incorrect origins, and a new species of adulterant was also identified. These results confirmed the accuracy and efficiency of the designed DNA markers; this method may be used as an efficient tool for the identification and verification of Amomi Fructus.

## 1. Introduction

Amomi Fructus, which is derived from species of cardamom plants, is an important traditional medicine used for curing digestive diseases, rheumatism, malaria, toothache, eliminating dampness, and promoting appetite [1,2]. It is also used as an ingredient in the production of cosmetic and food products. The ripe fruits from *Amomum villosum* Lour., *A. villosum* var. *xanthioides* Wall. ex Baker, T.L.Wu and S.J.Chen, and *A. longiligulare* T.L.Wu are known as the origin of Amomi Fructus [1]. However, there are differences in the definition of original species of Amomi Fructus among pharmacopeias of Korea, China, and Taiwan [3]. Amomi Fructus originates from the fruits of *A. villosum* and *A. villosum* var. *xanthioides* in Korean pharmacopeia [4], while the fruits of *A. longiligulare* in addition to those two species are listed as Amomi Fructus in both Chinese and Taiwan pharmacopeias [5,6]. Previous studies have reported that the content of borenol acetate as an active ingredient was significantly higher in the fruit of *A. villosum* than in *A. villosum* var. *xanthioides* and *A. longiligulare*, which explains the better therapeutic effect of *A. villosum* [7,8]. 

However, Amomi Fructus is frequently adulterated with the products of other *Amomum* species and even species from the genus *Alpinia* in the commercial herbal market [9,10,11]; over fifteen species in the genera *Amomum* and *Alpinia* have been reported as adulterants owing to their similar morphological and anatomical characteristics [11]. Moreover, similarities in morphological appearance also makes macroscopic distinction of the three genuine *Amomum* species much more difficult as they show few morphological differences [12]. 

Hence, accurate and reliable methods for the identification of Amomi Fructus are necessary to ensure quality and safety in medication, which can be guaranteed by using genuine products and avoiding adulterants. DNA barcoding is known as an efficient and accurate method of identifying species based on nucleotide diversity in short DNA segments. In several previous studies, samples of Amomi Fructus were genetically identified based on various DNA analytic techniques such as inter simple sequence repeats(ISSR)-PCR [13], random amplification of polymorphic DNA (RAPD) [14] and DNA barcoding, such as internal transcribed spacer (ITS) 1, ITS 2 and ITS including 5.8s rDNA and some plastid loci [15,16]. From previous studies, the DNA barcoding method is considered as a useful and reliable molecular tool for the identification of Amomi Fructus and its adulterants. The chloroplast (cp) genome has been established as the proper region for the DNA barcode for identifying the species in plants, especially between closely related species [17].

Therefore, in the present study, we used DNA barcoding analysis using five regions (ITS, *matK, rbcL, rpoB*, and *trnL-F* intergenic spacer) for genetic identification and evaluation of Amomi Fructus and its adulterants. Furthermore, we attempted to provide the DNA marker for the correct origins of Amomi Fructus based on the determined nucleotide sequences using the results of DNA barcoding analysis in this study.

## 2. Results

### 2.1. DNA Barcode Analysis

#### 2.1.1. Internal Transcribed Spacer (ITS) Regions of Nuclear Ribosomal Cistron

**Figure 1:**
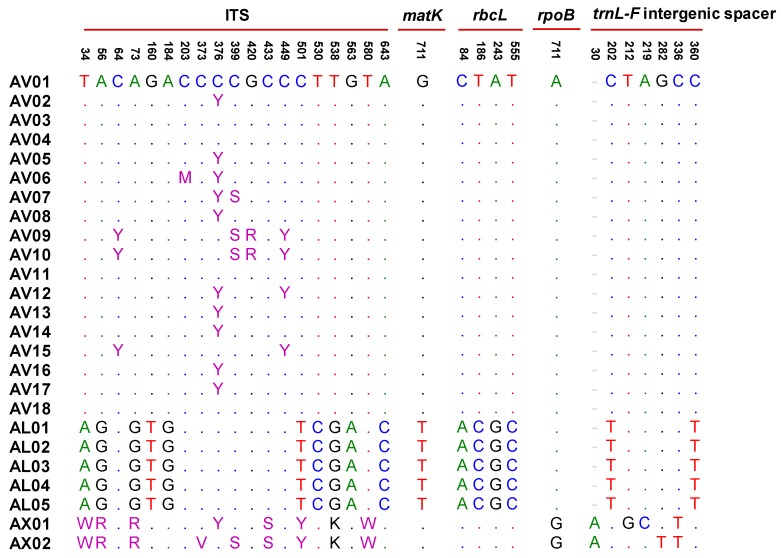
Multiple alignments of the 32 single nucleotide polymorphisms (SNPs) from five regions (ITS, *matK, rbcL, rpoB, trnL-F* intergenic spacer) in Amomi Fructus. Numbers above the bases indicate the position of single-nucleotide polymorphisms in each region. The dots indicate the consensus nucleotide; Sample code shown in Table 1. Heterozygous sites were defined according to IUPAC. AX01 (*A. villosum* var. *xanthioides*): KJ151892 and MH161417; AX02 (*A. villosum* var. *xanthioides*): KJ151893 and MN067432.

**Figure 2:**
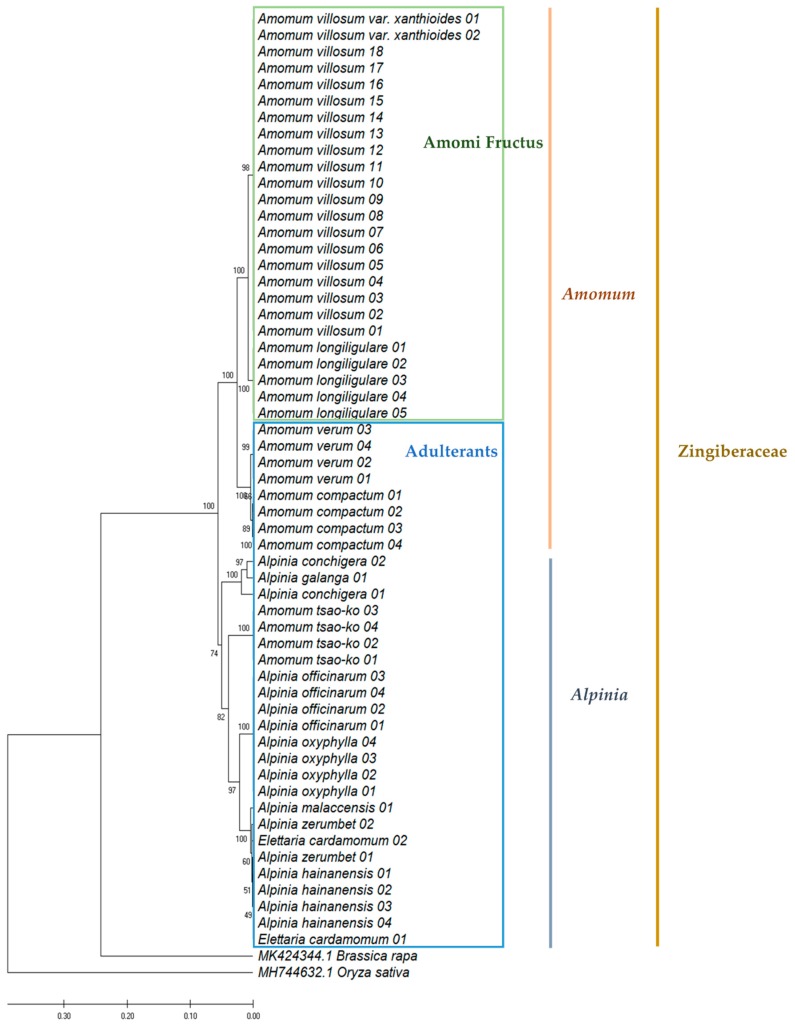
Phylogenetic analysis of Amomi Fructus and its adulterants based on the nucleotide sequences of internal transcribed spacer (ITS), including 5.8S rDNA region using the UPGMA method. The percentage of replicate trees in which the associated taxa clustered together in the bootstrap test (500 replicates) are shown next to the branches. The evolutionary distances were computed using the Maximum Composite Likelihood method and are in the units of the number of base substitutions per site. As outgroups, ITS nucleotide sequences of *Brassica rapa* (MK424344.1) and *Oryza sativa* (MH744632.1) were used.

To identify Amomi Fructus from several possible adulterants, the nucleotide sequences of the ITS region were analyzed. Approximately 645–665 bases of amplified product sequence was identified based on the samples listed in Table 1. The results of sequence characteristics were represented in Table 2. Among the original species of Amomi Fructus origin, 19 different nucleotides were observed from *A. villosum* var. *xanthioides*, *A. villosum*, and *A. longiligulare* (Figure 1). Although some intraspecific variations were detected in *A. villosum* species (AV01-AV18), these variations did not compromise the ability of distinguishing the original species of Amomi Fructus from the possible adulterants. The sequence identity matrix was 1 (maximum) to 0.974 (minimum) among the three species of Amomi Fructus (Table S1). The phylogenetic analysis showed that the samples of *A. villosum* formed a closer relationship with those of *A. villosum* var. *xanthioides* than with those of *A. longiligulare*. The sequence identity matrix ranged from 0.936–0.904 in adulterants of genus *Amomum*, while it ranged from 0.894 to 0.844 in adulterants of the genus *Alpinia*. The results of the phylogenetic analysis inferred through ITS nucleotide sequences showed that samples of genuine *Amomi* species formed a closer relationship and their groups were apparently distinguished from the other *Amomum* and *Alpinia* species which were recognized as adulterants (Figure 2). 

#### 2.1.2. Chloroplast Genome Based DNA Barcode Sequence Analysis

Four chloroplast genome areas were analyzed to determine the proper DNA barcode for Amomi Fructus and the adulterants. Sequence characteristics of the four plastid loci and one nuclear region are given in Table 2. The amplified product size varied from 395 bases (*trnL-F* intergenic spacer) to 933 bases in *matK*. The *rbcL* and *trnL-F* intergenic spacer regions have higher variable sites among four plastid loci (except ITS region). The sequences of *matK* and *rpoB* were the most conserved among the five regions analyzed when the aligned length and number of conserved sited were taken into consideration. Even though the variable site of four plastid loci were much lower than ITS region and highly conserved, it could separate the species of Amomi Fructus from adulterants. 

For more detail about the four plastid loci, the 933 bases partial nucleotide sequences of *matK* were determined using a 390F/1326R primer set. Unlike the ITS region, all of the samples showed the same nucleotide length, as 933 bases, that is, not only the genus *Amomum*, but also *Alpinia*. There was no intraspecific variation among the genuine species of Amomi Fructus. Further, the sequence identity matrix between Amomi Fructus and the adulterants was considerably closer as compared to the results with ITS; the minimum identity matrix result was 0.981 between Amomi Fructus and the adulterants (Table S2). As shown in Figure 1, no difference of nucleotide sequence was detected between *A. villosum* and *A. villosum* var. *xanthioides*, while only a one base difference was detected in *A. longiligulare* (Figure 1). Nevertheless, the phylogenetic tree analyzed by *matK* indicated that the original species of Amomi Fructus were well separated from the adulterants (Figure S1A). 

For the *rbcL* gene sequence, we identified a 743 bases partial nucleotide sequence in all the samples listed in Table 1 using the rbcL a-f/724R primer set. Four SNPs were observed among *A. villosum, A. villosum* var. *xanthioides* and *A. longiligulare* (Figure 1). There was no difference of nucleotide sequence between *A. villosum* and *A. villosum* var. *xanthioides*. The difference in the nucleotide sequence was lower than the ITS results; however, the three species of Amomi Fructus origin could still be distinguished. Although the minimum sequence identity matrix of *rbcL* among samples in Table 1 was near 0.986 (except in the ATK samples, where the minimum sequence identity matrix was 0.896), Amomi Fructus was well separated from the adulterants according to the results of the phylogenetic relationship analysis based on the *rbcL* nucleotide sequences (Table S2, Figure S1B).

In total, 516 bases partial nucleotide sequences were determined for the *rpoB* gene from the samples in Table 1. There was no difference in the nucleotide sequence between *A. villosum* and *A. longiligulare,* while only a one base difference was observed in *A. villosum* var. *xanthioides* (Figure 1). Only 16 SNPs were observed among the Amomi Fructus and several adulterants at *rpoB* gene (data not shown). The minimum sequence identity matrix among the samples in Table 1 was very close to 0.988. Thus, the Amomi Fructus samples were distinguished from the adulterant samples, except for AK and AC samples (Table S2, Figure S1C). 

In the case of the *trnL-F* intergenic spacer, 395–415 bases amplified products were determined from the samples listed in Table 1. The species of origin of Amomi Fructus were determined to be the 407–408 bases nucleotide sequences. Two base differences were observed between *A. villosum* and *A. longiligulare*. In the case of *A. villosum* var. *xanthioides*, intraspecific variations were observed between two previously deposited nucleotide sequences in NCBI Genbank (AX01-02). Seven bases differences, including intraspecific variations, were observed in *A. villosum* var. *xanthioides* (Figure 1). However, the amplified product size was different depending on the species and the sequence identity matrix between Amomi Fructus and the adulterants ranged from 0.975 to 0.882 (Table S2). As with the above three barcode regions, the phylogenetic tree inferred from the *trnL-F* intergenic spacer also showed distinguishable groups of Amomi Fructus samples from several adulterants (Figure S1D).

### 2.2. DNA Marker for Amomi Fructus Based on Discrepancy in the ITS Sequences

**Figure 3:**
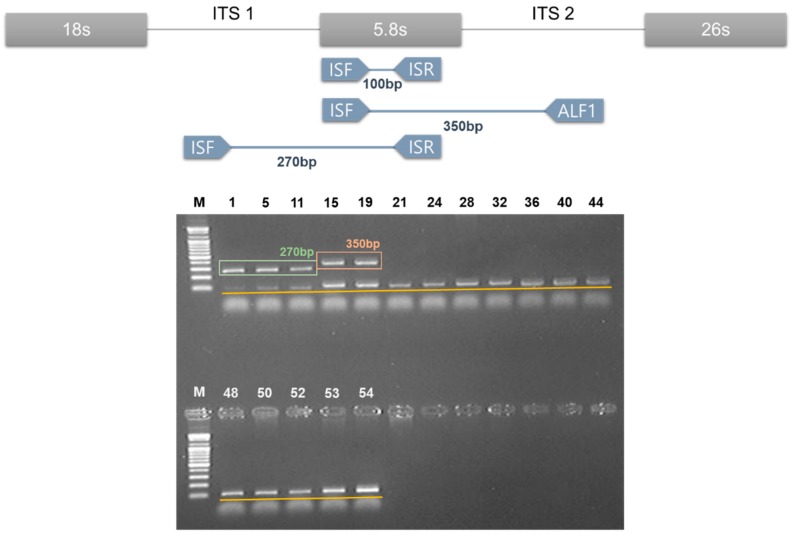
Multiplex PCR products of the primer set AVF1/ISR/ISF/ALF1 from randomly chosen samples in Table 1 for distinguishing Amomi Fructus from its adulterants. Lane numbers above: The sample number listed in Table 1. M: 100 bases ladder size marker.

From the results of five DNA barcode nucleotide sequences as determined above, we confirmed that those five DNA barcode regions were suitable to differentiate genuine Amomi Fructus samples from their adulterants. However, while DNA barcoding was an efficient method to identify the origin of each medicinal herb, sequencing five barcode regions would be a time-consuming process for monitoring medicinal herbs distributed in the commercial market. Therefore, we attempted to develop a DNA marker for the clear and quick distinction of Amomi Fructus from adulterants based on the determined nucleotide sequences in this study. In order to design the DNA marker, ITS sequences were chosen due to their high level of interspecific variations compared to the cpDNA barcodes. As shown in Figure 3, AVF1/ISR primer sets were developed as a DNA marker for *A. villosum*, which produced a 270 bases amplified product for *A. villosum* (Figure 3). ALF1/ISF primer sets, which produced a 350 bases amplified product, were developed as an *A. longiligulare*-specific DNA marker (Figure 3). In order to confirm the PCR reaction and rule out PCR error, an ISF/ISR primer set was designed as an internal standard marker. Also, we tried to multiplex the PCR process by designing three primer sets to improve the efficiency, and to save time and costs. One PCR reaction with AVF1/ISR/ISF/ALF1 marker produced 270 bases and 100 bases PCR products in *A. villosum* species, while 350 bases and 100 bases PCR products were produced by *A. longiligulare* species (Figure 3). All the other samples except Amomi Fructus produced 100 bases PCR products as the internal standard.

### 2.3. Monitoring Amomi Fructus Vouchers in Commercial Markets Using DNA Markers 

**Figure 4:**
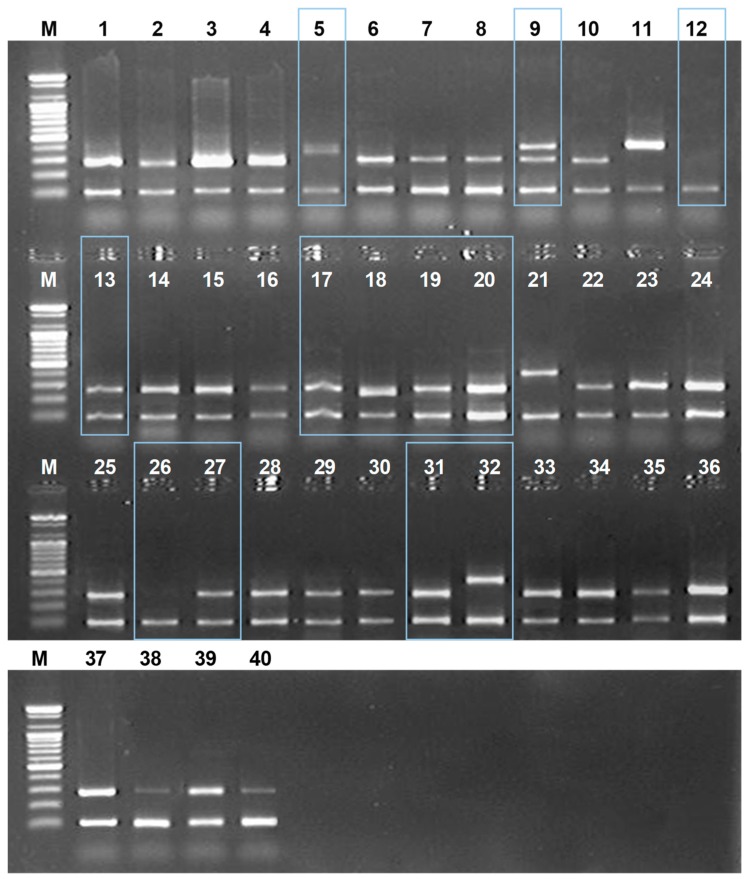
The monitoring results of commercial Amomi Fructus by using multiplex PCR with the designed AVF1/ISR/ISF/ALF1 primer set in this study. Lane numbers above: The sample number as listed in Table 2. M: 100 bases ladder; Box shows different amplified results than expected.

For the verification of the developed DNA markers, we collected and monitored 40 commercial samples sold as Amomi Fructus from various regions in the eastern and southern Asian countries (Table 3). We applied newly-developed DNA markers to identify commercial Amomi Fructus samples and thereafter confirmed their taxonomic affiliation using ITS nucleotide analysis. As shown in Figure 4, identified species of some commercial Amomi Fructus samples were not consistent with their originated species: (1) Some of the commercial *A. villosum* samples did not amplify with the AVF1/ISR primer set, but rather amplified with the ALF1/ISF, a specific DNA marker for *A. longiligulare*; (2) some of the commercial *A. longiligulare* samples were not amplified with the specific DNA marker set. All samples were amplified with ISF/ISR (internal standard marker), which indicates that there were no errors in the PCR reaction. 

We finally confirmed the taxonomic affiliation of commercial Amomi Fructus samples using ITS nucleotide sequence analysis (Table 3). Most of the commercial *A. villosum* samples were genetically re-identified as they were named. However, some samples named as *A. villosum* (No. 5 and No. 9) were re-identified as *A. longiligulare* and vice versa (No. 13 and 27). Sample No. 9 was mixed with *A. longiligulare.* Two samples (No. 12 and 26) that were named as *A. longiligulare* did not amplify with AVF1/ISR/ISF/ALF1 DNA marker and were re-identified as *A. ghaticum* K.G.Bhat, which was not previously reported as an adulterant of Amomi Fructus by using ITS nucleotide analysis combined with NCBI Blast result. All samples named as *A. villosum* var. *xanthioides* were re-identified as *A. villosum* (No. 17–20, 31) or *A. longiligulare* (No. 32). These results demonstrate that genetic identifications of commercial Amomi Fructus samples using developed DNA markers were consistent with those using the ITS nucleotide analysis. 

## 3. Discussion

DNA barcoding has been developed as a rapid and reliable technique to identify species based on variations in the sequence of short standard DNA region(s) [19,20,21,22,23,24]. This tool is successfully used in a variety of biological applications, including discovering cryptic species, detecting invasive species, and reconstructing food webs [17]. Furthermore, it has been widely accepted as a technique to authenticate herbal medicinal materials (e.g., powder, processed roots, barks, and leaves) and in the detection of product substitution and contamination [25,26,27,28]. Unfortunately, it is very common for morphologically similar herbs to be used as adulterants in commercial herbal markets. Although the authentication of closely related species using DNA barcoding has been challenging, DNA barcoding can readily distinguish species that are morphologically similar but phylogenetically different [29]. 

In previous research on the identification of Amomi Fructus, DNA barcoding using the novel SNPs substantiated its effectiveness [1,9,10,11,15,16,30]. Based on these studies, we selected five DNA barcoding regions, that is, ITS, *rbcL, matK, rpoB*, and *trnL-F* intergenic spacer, to distinguish Amomi Fructus from its adulterants. 

Among the original species of Amomi Fructus, the nuclear ribosomal DNA ITS region showed more SNPs than the plastid. The minimum sequence identity matrix of genuine Amomi Fructus samples showed that the ITS is more variable than the plastid regions. These results indicate that the ITS region has high levels of sequence differentiation among the species and shows greater discriminatory power than the plastid region.

**Figure 5:**
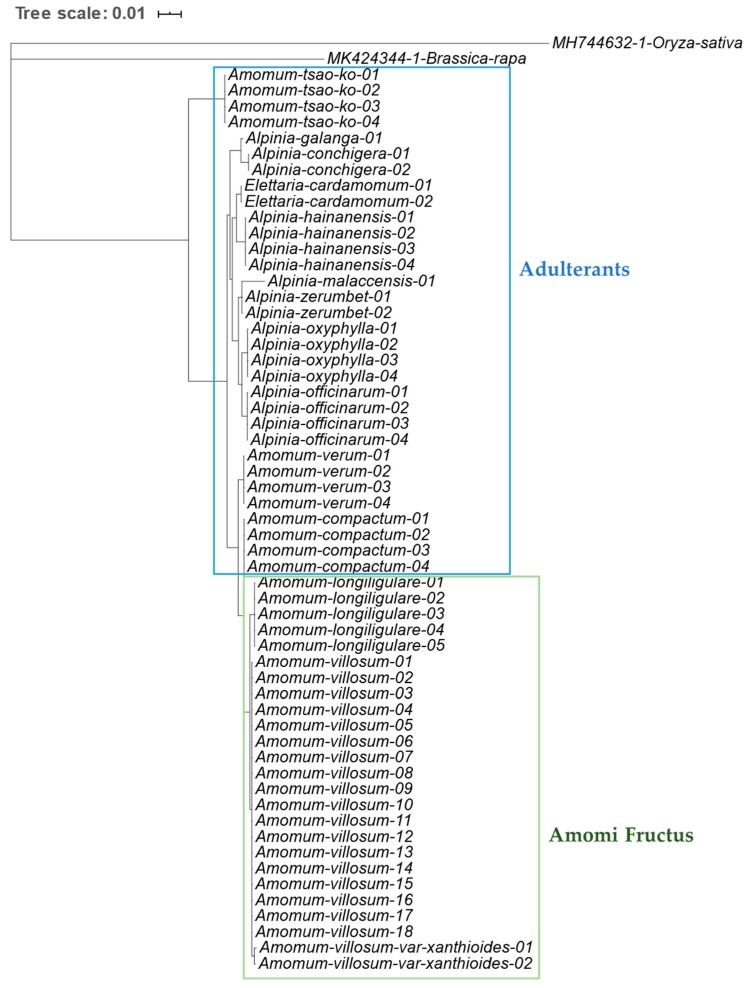
Phylogenetic analysis of Amomi Fructus and its adulterants based on the concatenate nucleotide sequences of four plastid regions (*matK, rbcL, rpoB* and *trnL-F* intergenic spacer) using the MrBayes.

Despite the high discriminatory efficiency of ITS, a single region may be insufficient to cover the various varieties [17], and it sometimes shows analytical problems in samples contaminated by fungi. Thus, multiple loci are necessary for the maximal identification and prevention of the fungal contamination problem. Firstly, we analyzed the CBOL-Plant Working Group recommendation of two-locus barcoding combination *rbcL* + *matK* [21]. The *rbcL* is one of the large subunits which encode the critical photosynthetic enzyme rubilose-1-5-bisphosphate carboxylase/oxygenase (RUBISCO), the first sequenced gene from plants [31]. The *matk* is also known as one of the most rapidly evolving genes and has been used as a marker to construct plant phylogenies [32,33,34]. This two-locus combination was expected as the universal barcode for land plants [21]. The *rbcL* + *matK* combination shows a total of six SNPs among the original species of Amomi Fructus. The variation rate was lower than ITS and *matK* alone was not suitable for the identification of Amomi Fructus. However, an *rbcL* plus *matK* combination could successfully separate Amomi Fructus from the adulterants (data not shown). Even if this combination was a proper locus, the amplification size could be long for manufactured samples by various processing methods. Therefore, we tried to analyze additional locus *rpoB* and *trnL-F* intergenic spacer, which are shorter. At first, we analyzed the *trnH-psbA* locus, which is one of the most variable genome segments in the chloroplast of angiosperms [24], instead of *rpoB*. It was short and easily amplified in any species, but we had to eliminate this locus because of several non-specific indels. The *rpoB* gene encodes the subunit of the chloroplast RNA polymerase and is currently considered as the core gene for DNA barcoding in bacteria [17] rather than plants. However, it has high universality and it yields high-quality sequences [17], a number of which have already been deposited in NCBI GenBank. Therefore, we chose the *rpoB* instead of *trnH-psbA*, even though it was not recommended by CBOL-Plant Working Group. *trnL-F* intergenic spacer has been used for molecular phylogenetic studies of various taxa since it was introduced by Taberlet et al. [35]. These two regions were also not quite successful as single-locus, but separated the samples followed by the species when combined together (data not shown). The results of the DNA barcoding of the plastid regions confirmed that the combination of the two regions is more efficient than the single region alone. Therefore, we combined all four plastid regions to perform the phylogenetic analysis using MrBayes (Figure 5). Concatenating the four plastid regions allowed the successful identification of each authentic species of Amomi Fructus and efficiently separated the adulterants. 

Our results from DNA barcode analysis confirmed the ability of the classification for Amomi Fructus and the adulterants at the species level. Recently, Cui et al. reported the complete genome of *A. villosum*, *A. villosum* var. *xanthioides* and *A. longiligulare* via high-throughput sequencing [12]. They found five new divergent regions (*atpH-atpI, trnD-trnY, accD-psaI, ycf4-cemA and trnI-ycf2*), which were not useful as effective molecular markers. However, this was the first approach to sequence and determine the complete complete genome for Amomi Fructus. Thus, we used these deposited complete genome data as a reference in this work and further studies.

In addition, we attempted to provide the species-specific DNA marker to determine the origin of Amomi Fructus. DNA barcoding is a very powerful method to identify species; however, it is time-consuming and not cost-effective for monitoring distributed samples. Therefore, we tried to develop the DNA marker for Amomi Fructus. The SNP-based DNA Marker has been a successful method to identify the medicinal herbs from its adulterants [36,37,38,39,40]. In the case of Amomi Fructus, the recoded origin species in pharmacopoeia was different according to the country of origin. Two species, *A. villosum* var. *xanthioides* and *A. villosum*, were recorded in the Korean Pharmacopoeia [4]. *A. longiligulare* as well as the above two species are additionally registered in Chinese and Taiwan pharmacopoeias [5,6]. Therefore, the fruits of *A. longiligulare* should not be used as herbal medicine in Korea. In this study, we tried to develop one common DNA markers for *A. villosum* and *A. villosum* var. *xanthioides* and one marker for *A. longiligulare*. Unfortunately, we could not collect correctly identified *A. villosum* var. *xanthioides* samples and, therefore, we used the NCBI GenBank Database for Amomi Fructus instead, as previously reported [1,12]. To confirm any PCR reaction errors, we designed the internal standard marker based on 5.8S ribosomal DNA region in ITS. As shown in Figure 4, the designed DNA marker provided reliable differentiation results. 

With the developed DNA markers, we monitored commercial Amomi Fructus samples in herbal markets in Korea and other Asian countries. To confirm the DNA marker result, we analyzed the ITS nucleotide sequences together. As shown in Figure 5, misuse existed in some samples. Some of them were distributed with incorrect medicinal names, such as ‘*A. longiligulare*’ to ‘*A. villosum*’, ‘*A. villosum*’ to ‘*A. villosum* var. *xanthioides*’ and ‘*A. longiligulare*’ to ‘*A. villosum* var. *xanthioides*’. Furthermore, we found a previously unreported new adulterant of Amomi Fructus. Some of the *A. longiligulare* samples were identified as *Amomum ghaticum* through the blast analysis in NCBI GenBank data. *A. ghaticum* was originally published in Indian J. Forest. 11: 322 1988 publ. 1989 [41]. *A. ghaticum* has not been reported as an adulterant and is known as an endemic plant in the Western Ghats of India [42,43]; therefore, further research will be necessary. In this study, we confirmed the ability of DNA barcoding analysis for the identification of Amomi Fructus and its adulterants and suggested proper regions for classified the Amomi Fructus. Moreover, we have presented a species-specific DNA marker for the identification of Amomi Fructus. This may be a useful tool to reduce the time and cost involved in monitoring and confirming the quality Amomi Fructus for commercial usage.

## 4. Materials and Methods 

### 4.1. Plant Materials

For the identification of species of Amomi Fructus and its adulterants, fifty-five samples of crude drugs and voucher specimens of Amomi Fructus were collected or purchased from the habitats, cultivation areas, and local markets in Korea, China, and Myanmar (Table 1). Some samples were received from the Korean institute of Oriental Medicine. For monitoring Amomi Fructus samples in the Korean commercial markets, a total of forty crude drug samples were tested (Table 3). All the samples and isolated genomic DNA were deposited at the herbarium of Korean Medicine in Wonkwang University. 

### 4.2. Preparation of Genomic DNA

The genomic DNA was extracted from the Amomi Fructus according to the manual of NucleoSpin^®^ Plant II kit (Macherey-Nagel, Dueren, Germany) with PL1 lysis buffer. For some samples, 10% cetyltrimethyl ammonium bromide (CTAB) and 0.7M NaCl were used to remove the phenolic compounds and polysaccharides. 

### 4.3. PCR Amplification for DNA Barcode Analysis 

For ITS amplification, PCR was performed using T-personal cycler (Biometra, Germany). In brief, 600 nM of primer set of ITS1 (5″-TCCGTAGGTGAACCTGCGG-3′) and ITS4 (5′-TCCTCCGCTT ATTGATATGC-3′) [44], 1X AccuPower^®^ GoldHotStart Taq PCR PreMix (Bioneer, Daejeon, Korea), and a 30 ng of genomic DNA were used for PCR amplification. PCR cycling conditions followed by a pre-denaturation process (95 °C, 5 min) were as follows: Denaturation process (95 °C, 30 s); annealing process (52 °C, 30 s); extension process (72 °C, 40 s) × 36 cycles; final extension process (72 °C, 5 min). For chloroplast DNA barcoding regions, rbcL a-f (5′-ATGTCACCACAAACAGAGAC TAAAGC-3′)/724R (5′-TCGCATG TACCTGCAGTAGC-3′) and 390F (5′-CGATCTATTCATTCAAT ATTTC-3′)/1326R (5′-TCTAGCACAC GAAAAGTCGAAGT-3′) primer sets were used for amplification of *rbcL* and *matK* regions [24,45,46]. rpoB1 (5′-AAGTGCATTGTTGGAACTGG-3′)/rpoB3 (5′-CCGTAT GTGAAAAGAAGTATA-3′) and trnL-e (5′-GGTTCAAGTCCCTCTTATCCC-3′)/trnL-f (5′-ATTTGA ACTGGTGACACGAG-3′) primer sets were used for *rpoB* and *trnL-F* intergenic spacer regions [23,35]. The amplified PCR product was separated from other gradients using 1.5% agarose gel electrophoresis after staining by the addition of Safe-whiteTM (abm, Richmond, Canada). Amplified products were analyzed using MyImage (Seoulin Biotechnology, Seongnam, Korea). 

### 4.4. Determination of DNA Sequences of PCR Product

PCR products separated from agarose gel were cloned using TOPcloner™ TA Kit (Enzynomics, Daejeon, Korea) and the DNA sequences of cloned PCR product were determined through the interpretation performed by Bioneer (Daejeon, Korea). For accuracy, the DNA barcode analysis process was performed independently three times from the preparation of the genome DNA stage.

### 4.5. Analysis of DNA Sequences and Preparation of Dendrogram

DNA sequences were analyzed using ClustalW multiple sequence alignment (Bioedit, v7.0.9; available from http://www.mbio.ncsu.edu/BioEdit/page2.html) and confirmed with multiple sequence alignment in MAFFT (MAFFT, v7; available from https://mafft.cbrc.jp/alignment/server/). To verify the polymorphisms, represented by IUPAC symbols in the sequence data, all sequences were generated at least twice. The chromatograms of nucleotide sequences, which were provided from Bioneer sequencing service, were compared. Evolutionary analyses were conducted in MEGA X (v10.0.5; available from https://www.megasoftware.net/). Phylogenic trees were constructed for each region using distance-based method (UPGMA and NJ) and the evolutionary distances in tree were computed using the Maximum Composite Likelihood method. Phylogenetic analysis of concatenate four plastid regions was constructed using MrBayes (MrBayes, 3.2.6_1; available from https://ngphylogeny.fr/tools/tool/281/form). All the analyzed sequences were compared with NCBI GenBank using BLAST (Altschul et al. 1990) [47]. Newly determined nucleotide sequences were deposited in NCBI GenBank. As the outgroups, we used *Brassica rapa* (MK424344.1) and *Oryza sativa* (MH744632.1) deposited in the NCBI GenBank [48].

### 4.6. Multiplex PCR

For the multiplex PCR amplification with developed DNA markers, each 0.5 pmol of the primers AVF1 (5′-TGGATGATTGTGAACGTGTCAACA-3′) and ALF1 (5′-AGGGTCTCTTTGAGGACACAT CCC G-3′), each 0.5 pmol of the primers ISR (5′-AAAGACTCAATGGTTCACGAG-3′) and ISF (5′-GACTCTCGGCAATGGATATCT-3′); 1X AccuPower^®^ GoldHotStart Taq PCR PreMix (Bioneer, Daejeon, Korea), and a 20 ng of genomic DNA were used for PCR amplification. During the 23-cycle PCR process, pre-denaturation was conducted for 5 min at 95 °C and denaturation for 30 s at 95 °C. The annealing process was conducted for 10 s at 58.5 °C and the extension process for 20 s at 72 °C. A final reaction step was conducted for 5 min at 72 °C. The amplified products were separated on 2% agarose gel and revealed by staining with Safe-whiteTM (abm, Richmond, Canada). The specific amplified regions by developed DNA marker were confirmed by analysis of nucleotide sequences. 

## 5. Conclusions

The purpose of this study was to provide efficient and proper methods to identify and monitor the origin of Amomi Fructus, which has an increased usage not only in commonly used foods and drugs but also cosmetic ingredients. Moreover, adulterant problems frequently occurred due to morphological similarity. We suggested proper DNA barcode regions to distinguish the Amomi Fructus from the adulterants. Furthermore, we designed an SNP-based DNA marker for Amomi Fructus. The monitoring results using multiplex PCR with AVF1/ISR/ISF/ALF1 DNA markers successfully identify the species in Amomi Fructus. We hope that our approach will prove a useful tool to reduce the time and cost of monitoring and confirming the quality of Amomi Fructus for commercial usage.

## Figures and Tables

**Table 1:** The collected Amomi Fructus and Zingiberaceae plants samples used in this study.

No.	Sample Code	Scientific Name	Medicinal Name
**1**	AV01	*Amomum villosum* Lour. (=*Wurfbainia villosa* (Lour.) Skornick. A.D.Poulsen)	Amomi Fructus*,**
**2**	AV02		
**3**	AV03		
**4**	AV04		
**5**	AV05		
**6**	AV06		
**7**	AV07		
**8**	AV08		
**9**	AV09		
**10**	AV10		
**11**	AV11		
**12**	AV12		
**13**	AV13		
**14**	AV14		
**15**	AV15		
**16**	AV16		
**17**	AV17		
**18**	AV18		
**19**	AL01	*Amomum longiligulare* T.L.Wu (=*Wurfbainia longiligularis* (T.L.Wu) Skornick. A.D. Poulsen)	Amomi Fructus**
**20**	AL02		
**21**	AL03		
**22**	AL04		
**23**	AL05		
**24**	AK01	*Amomum verum* Blackw. (=*Amomum krervanh* Pierre ex Gagnep.)	Amomi Fructus Rotundus
**25**	AK02		
**26**	AK03		
**27**	AK04		
**28**	AC01	*Amomum compactum* Sol. ex Maton	
**29**	AC02		
**30**	AC03		
**31**	AC04		
**32**	ATK01	*Amomum tsao-ko* Crevost Lemarié (=*Amomum tsaoko*)	Amomi Tsao-ko Fructus
**33**	ATK02		
**34**	ATK03		
**35**	ATK04		
**36**	AH01	*Alpinia hainanensis* K.Schum. (=*Alpinia katsumadae* Hayata)	Alpiniae Katsumadai Semen
**37**	AH02		
**38**	AH03		
**39**	AH04		
**40**	AO01	*Alpinia oxyphylla* Miq.	Alpiniae Oxyphyllae Fructus
**41**	AO02		
**42**	AO03		
**43**	AO04		
**44**	AOR01	*Alpinia officinarum* Hanc	Alpiniae Officinari Rhizoma
**45**	AOR02		
**46**	AOR03		
**47**	AOR04		
**48**	ACC01	*Alpinia conchigera* Griff.	jie bian shan jiang***
**49**	ACC02		
**50**	AZ01	*Alpinia zerumbet* (Pers.) B.L.Burtt R.M.Sm.	yan shan jiang***
**51**	AZ02		
**52**	AM01	*Alpinia malaccensis*(n.l.Burman) Roscoe	mao ban shan jiang***
**53**	AG01	*Alpinia galanga* (l.) Willd.	Galangae Fructus
**54**	EC01	*Elettaria cardamomum* (l.) Maton (=*Amomum cardamomum* l., *Alpinia cardamomum* (l.) Roxb.)	Cardamomi Fructus
**55**	EC02		

*: The Korean Pharmacopoeia, 11th edition; **: Pharmacopoeia of the Peoples Republic of China, Taiwan Herbal Pharmacopeia; ***: Flora of China [18].

**Table 2:** Amplicon size of plastid loci and nuclear barcode region in Amomi Fructus and adulterant species and the sequence characteristic, single and in different multi-region combination.

Barcode Target	Amplicon Size (~Bases)	Aligned Length (Bases)	Conserved Sites	Variable Sites	Parsimony Informative Sites	Singleton Sites
ITS	645–665	670	516	154	132	22
*matk*	933	933	894	39	27	12
*rbcL*	743	743	657	86	12	74
*rpoB*	516	516	500	16	10	6
*trnL-F* intergenic sapcer	395–415	422	377	43	13	30
*matk* + *rbcL*		1676	1551	125	39	86
*rpoB* + *trnL-F* intergenic spacer		938	877	59	23	36
*mark* + *rbcL* + *rpoB*		2192	2051	141	49	92
*mark* + *rbcL* + *trnL-F* intergenic spacer		2098	1928	168	52	116
Four plastid targets		2614	2428	184	62	122

**Table 3:** The re-identification results of collected monitoring samples based on ITS nucleotide sequence analysis.

No	Collected Species Name	Genetically Re-Identified Species	No	Collected Species Name	Genetically Re-Identified Species
**1**	*A. villosum*	*A. villosum*	**21**	*A. longiligulare*	*A. longiligulare*
**2**	*A. villosum*	*A. villosum*	**22**	*A. villosum*	*A. villosum*
**3**	*A. villosum*	*A. villosum*	**23**	*A. villosum*	*A. villosum*
**4**	*A. villosum*	*A. villosum*	**24**	*A. villosum*	*A. villosum*
**5**	*A. villosum*	*A. longiligulare*	**25**	*A. villosum*	*A. villosum*
**6**	*A. villosum*	*A. villosum*	**26**	*A. longiligulare*	*Amomum ghaticum*
**7**	*A. villosum*	*A. villosum*	**27**	*A. longiligulare*	*A. villosum*
**8**	*A. villosum*	*A. villosum*	**28**	*A. villosum*	*A. villosum*
**9**	*A. villosum*	*A. villosum/A. longiligulare*	**29**	*A. villosum*	*A. villosum*
**10**	*A. villosum*	*A. villosum*	**30**	*A. villosum*	*A. villosum*
**11**	*A. longiligulare*	*A. longiligulare*	**31**	*A. villosum* var. *xanthioides*	*A. villosum*
**12**	*A. longiligulare*	*Amomum ghaticum*	**32**	*A. villosum* var. *xanthioides*	*A. longiligulare*
**13**	*A. longiligulare*	*A. villosum*	**33**	*A. villosum*	*A. villosum*
**14**	*A. villosum*	*A. villosum*	**34**	*A. villosum*	*A. villosum*
**15**	*A. villosum*	*A. villosum*	**35**	*A. villosum*	*A. villosum*
**16**	*A. villosum*	*A. villosum*	**36**	*A. villosum*	*A. villosum*
**17**	*A. villosum* var. *xanthioides*	*A. villosum*	**37**	*A. villosum*	*A. villosum*
**18**	*A. villosum* var. *xanthioides*	*A. villosum*	**38**	*A. villosum*	*A. villosum*
**19**	*A. villosum* var. *xanthioides*	*A. villosum*	**39**	*A. villosum*	*A. villosum*
**20**	*A. villosum* var. *xanthioides*	*A. villosum*	**40**	*A. villosum*	*A. villosum*

## Supplementary Materials

The following are available online. Table S1. The sequence identity matrix among the Amomi Fructus and its several adulterants based on the ITS nucleotide sequence. Table S2. The sequence identity matrix among the Amomi Fructus and its several adulterants based on the four DNA barcode loci in chloroplast genome. Figure S1. Neighbor-joining (NJ) tree of Amomi Fructus and its adulterants based on the nucleotide sequences of (A) *matK*, (B) *rbcL*, (C) *rpoB* and (D) *trnL-F* intergenic sapcer regions of sample listed in Table 1. As outgroups, *Brassica rapa* and *Oryza sativa* were used.
