# Supplementary figures and images for: Identification and Monitoring of Amomi Fructus and its Adulterants Based on DNA Barcoding Analysis and Designed DNA Markers
Source: Molecules. 2019 Nov 19;24(22):4193. doi: 10.3390/molecules24224193 (PMC6891445; doi:10.3390/molecules24224193)

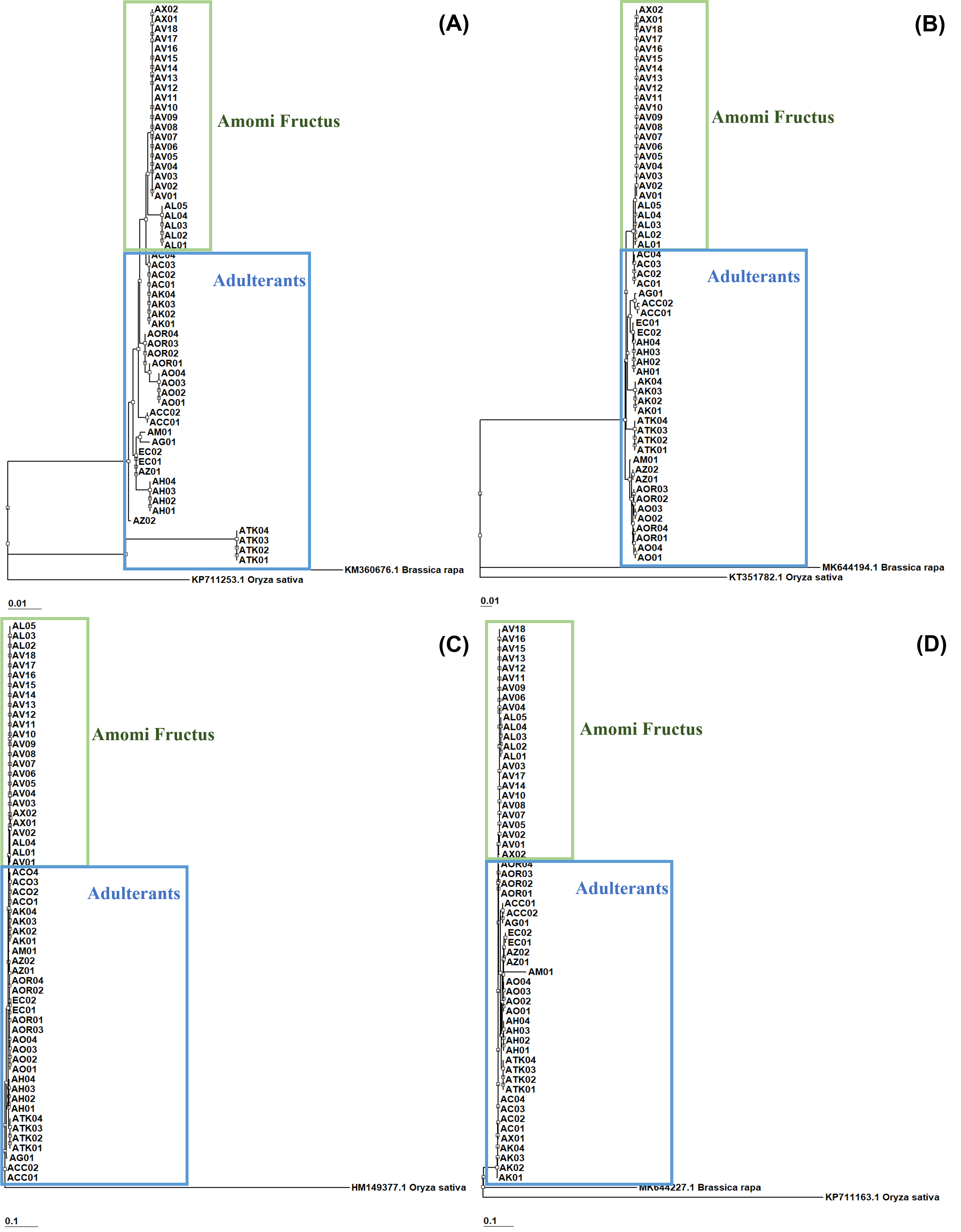

Supplement: Supplementary file 1 [file molecules-24-04193-s001.zip › molecules-632480-SI/Figure S1.png]
